# Biological determinants of functioning in euthymic patients with Bipolar Disorder: A multicentric 3-year cohort study

**DOI:** 10.1192/j.eurpsy.2022.576

**Published:** 2022-09-01

**Authors:** Y. Cañada, A. García-Blanco, P. Navalón, M. Sanchez Autet, L. De La Fuente Tomas, M.P. Garcia-Portilla, B. Arranz, P. Sierra San Miguel

**Affiliations:** 1 La Fe University and Polytechnic Hospital, Psychiatry, Valencia, Spain; 2 La Fe Health Research Insitute, Mental Health Research Group, Valencia, Spain; 3 La Fe Health Research Institute, Neonatal Research Group, Valencia, Spain; 4 Parc Sanitari San Joan de Deu, Psychiatry, Barcelona, Spain; 5 University of Oviedo, Department Of Psychiatry, Oviedo, Spain; 6 Centre for Biomedical Research Network on Mental Health (CIBERSAM), Instituto De Salut Carlos Iii, Madrid, Spain; 7 University of Oviedo, Department Of Psychiatry, oviedo, Spain

**Keywords:** bipolar disorder, Sexual functioning, sleep, functioning

## Abstract

**Introduction:**

Bipolar disorder is related with functional impairment in euthymia. The contribution of biological functions such as sleep, sexual functioning; or the presence of obesity on this loss remain understudied.

**Objectives:**

The aim of this work was to study the influence of biological determinants in context with clinical and demographical determinants of functioning in a 3-year cohort of euthymic BD patients.

**Methods:**

In this multicentric study 67 euthymic adult bipolar outpatients were followed during three years. Functioning was assessed with FAST, insomnia severity with Oviedo Sleep Questionnaire (OSQ) and, sexual functioning with Changes on Sexual Functioning Questionnaire (CSFQ-14) and obesity was expressed as body mass index (BMI). The basal effect of sleep, sexual functioning and obesity (Time 0) on FAST (Time 3) was analyzed with a mixed ordinal regression model including time effect, age, sex, number of manic and depressive episodes, euthymia length, and comorbidity with personality disorder. Change in functioning (Time 3 to 0) was analyzed in another mixed model also considering the difference in biological determinants (Time 3 to 0) and the presence of mood episodes during the period.

**Results:**

A basal worse sexual functioning, a higher severity of insomnia and a higher BMI predicted a worse functioning at three years (p=0.005, p=0.043, p=0.05 respectively). Regarding FAST difference from Time0 to 3, only having a manic episode related to an impairment on functioning (p=0.027).

**Conclusions:**

Sexual functioning, quality of sleep and BMI are predictors of functioning in euthymia in BD. Manic episodes in the following contribute to impairments on functioning more than depressive episodes.

**Disclosure:**

No significant relationships.

